# Metaverse and Medical Diagnosis: A Blockchain-Based Digital Twinning Approach Based on MobileNetV2 Algorithm for Cervical Vertebral Maturation

**DOI:** 10.3390/diagnostics13081485

**Published:** 2023-04-20

**Authors:** Omid Moztarzadeh, Mohammad (Behdad) Jamshidi, Saleh Sargolzaei, Fatemeh Keikhaee, Alireza Jamshidi, Shabnam Shadroo, Lukas Hauer

**Affiliations:** 1 Department of Stomatology, University Hospital Pilsen, Faculty of Medicine in Pilsen, Charles University, 323 00 Pilsen, Czech Republic; 2 Department of Anatomy, Faculty of Medicine in Pilsen, Charles University, 323 00 Pilsen, Czech Republic; 3 Faculty of Electrical Engineering, University of West Bohemia, Univerzitní 22, 306 14 Pilsen, Czech Republic; 4 Department of Computer Engineering, Mashhad Branch, Islamic Azad University, Mashhad 9187147578, Iran; 5 Department of Orthodontics, Faculty of Dentistry, Zahedan University of Medical Sciences, Zahedan 9816743463, Iran; 6 Dentistry School, Babol University of Medical Sciences, Babol 4717647745, Iran

**Keywords:** dental surgery, dentistry, digital twins, deep learning, CNN, advanced mathematical models, artificial intelligence, metaverse, healthcare

## Abstract

Advanced mathematical and deep learning (DL) algorithms have recently played a crucial role in diagnosing medical parameters and diseases. One of these areas that need to be more focused on is dentistry. This is why creating digital twins of dental issues in the metaverse is a practical and effective technique to benefit from the immersive characteristics of this technology and adapt the real world of dentistry to the virtual world. These technologies can create virtual facilities and environments for patients, physicians, and researchers to access a variety of medical services. Experiencing an immersive interaction between doctors and patients can be another considerable advantage of these technologies, which can dramatically improve the efficiency of the healthcare system. In addition, offering these amenities through a blockchain system enhances reliability, safety, openness, and the ability to trace data exchange. It also brings about cost savings through improved efficiencies. In this paper, a digital twin of cervical vertebral maturation (CVM), which is a critical factor in a wide range of dental surgery, within a blockchain-based metaverse platform is designed and implemented. A DL method has been used to create an automated diagnosis process for the upcoming CVM images in the proposed platform. This method includes MobileNetV2, a mobile architecture that improves the performance of mobile models in multiple tasks and benchmarks. The proposed technique of digital twinning is simple, fast, and suitable for physicians and medical specialists, as well as for adapting to the Internet of Medical Things (IoMT) due to its low latency and computing costs. One of the important contributions of the current study is to use of DL-based computer vision as a real-time measurement method so that the proposed digital twin does not require additional sensors. Furthermore, a comprehensive conceptual framework for creating digital twins of CVM based on MobileNetV2 within a blockchain ecosystem has been designed and implemented, showing the applicability and suitability of the introduced approach. The high performance of the proposed model on a collected small dataset demonstrates that low-cost deep learning can be used for diagnosis, anomaly detection, better design, and many more applications of the upcoming digital representations. In addition, this study shows how digital twins can be performed and developed for dental issues with the lowest hardware infrastructures, reducing the costs of diagnosis and treatment for patients.

## 1. Introduction

Determining a person’s growth status is crucial to many medical and dental procedures [1,2]. Specifically, before undergoing jaw surgery, placement of endosseous implants, or receiving a dental crown, one of the key factors that must be assessed is whether the patient has reached their full growth potential. While various approaches can be used to determine one’s growth status, examining skeletal indicators has been used extensively [3]. One method for evaluating skeletal maturity involves the examination of hand–wrist X-rays. However, lateral cephalometric radiographs, which are utilized for orthodontic diagnosis and treatment planning, also contain landmarks that can determine the stage of skeletal maturity. Therefore, lateral cephalometric radiographs offer a way to minimize unnecessary radiation exposure by eliminating the need for hand–wrist X-rays. This determination can be made through the use of the cervical vertebral maturation (CVM) method, which closely observes changes in the shape of the cervical vertebrae [4].

Artificial intelligence (AI) can be defined as a discipline within computer science that focuses on creating systems capable of performing tasks that require human-like intelligence. AI should have the ability to assess the information it receives, handle incomplete or incorrect information, and manage its sources [5]. Two key components of AI are learning and expanding its knowledge base. Machine learning (ML), a subfield of AI, trains algorithms using data rather than being explicitly programmed for a specific function. The artificial neural network (ANN) algorithm, a component of ML, is a mathematical model inspired by the way the biological nervous system processes information [6]. ANN strives to solve problems that involve the human-like abilities of thinking and observation. It consists of an input layer, intermediate hidden layers, and an output layer, with connections between processing units (neurons) that have numerical weights. Over time, the network adjusts these weights, allowing it to “learn”. There are two main ways to teach an AI model: supervised learning, where human experts label or extract features from the data, and unsupervised learning, where the features are automatically extracted by the algorithm without manual intervention.

The COVID-19 pandemic and the fourth industrial revolution are leading to the creation of a new and advanced form of digital civilization. In this modern society, corporations are the main connection between individuals and the community. The metaverse is now the driving force behind business opportunities in this digital world, and the COVID-19 situation has greatly impacted the metaverse-related business environment [7,8]. The traditional face-to-face business culture has given way to a zero-contact business culture, with online business becoming more prevalent across all industries. One of the sectors most affected by this shift is the healthcare industry, which has adopted smart technologies to provide services in a contactless manner. With the increase in the use of smart services and the expansion of their applications, it is clear that the zero-contact business culture has profoundly impacted the healthcare industry [9,10,11].

The metaverse is a developing technology that creates virtual environments where users can access a wide range of virtual services while enjoying immersive interactions with the real world. To bridge the gap between the virtual and real worlds, digital twins act as representatives of assets in this virtual space. By translating difficult assets, objects, and illnesses such as cancer into this digital world, patients can take advantage of its benefits [7,12]. The technology of blockchain is beneficial in the healthcare and metaverse industries because of its ability to create secure, decentralized, and transparent systems. It can be used in the metaverse to establish a virtual economy and in healthcare to secure data privacy and safety, track the source of pharmaceuticals, and simplify clinical trials. For instance, researchers proposed an architecture that used sophisticated encryption methods, such as RSA, ECC, and AES algorithms, to guarantee secure data transfer and storage while maintaining privacy in healthcare. This design aimed to support the decentralized storage and sharing of health data while keeping its confidentiality and integrity intact to fulfill the growing demand for privacy in scenarios where confidentiality is necessary. The proposed solution was created to address the increasing pace of blockchain technology [13].

The summary in Table 1 presents an overview of various papers having different topics and research methodologies that are relevant to the current paper. The evaluation of a paper’s introduction was based on its methodology and key findings, which were assessed for their relevance to the paper’s topic. In Korde et al.’s study, they reviewed different techniques for measuring skeletal maturity indicators and categorized traditional methods. Although the topic was different from the paper, the methodology of reviewing various techniques could be relevant if the paper needed to compare different methods, particularly in relation to the assessment of cervical vertebral maturation. Rolland-Cachera et al.’s research focused on growth parameters and their evolution based on references. While their findings may not have been directly applicable to the paper, the methodology of assessing growth parameters could be useful in certain situations. Understanding how to assess growth parameters could inform the paper’s approach to evaluating cervical vertebral maturation.

Fang et al. presented a hybrid approach to repair jaw defects based on concentrated growth factors. Although the subject matter was different, the clinical application of growth factors was relevant to the paper, and the methodology could provide insight into the application of growth factors. While the subject matter was different, the clinical application of growth factors was relevant to the paper, and the methodology could be useful for the paper’s approach to diagnosis and treatment. Ferrillo et al. conducted a systematic review to determine the reliability of cervical vertebral maturation. Although this subject matter may not have been relevant to the paper, the methodology of conducting a systematic review could be useful if the paper needed to review various studies related to cervical vertebral maturation assessment. Hamet et al. recommended different AI methods for different medical issues after assessing several methods. This methodology of assessing different methods could be relevant to the paper if it needed to evaluate different AI methods for medical diagnosis and treatment. Jamshidi et al. presented a hybrid echo state network for hypercomplex pattern recognition, classification, and big data analysis. Although the subject matter was different, the methodology of presenting a hybrid AI approach could be useful in certain situations. Jamshidi et al. created cancer digital twins using three ML methods. This methodology of using ML methods to create digital twins could be relevant to the paper if it needed to create a digital twin for a medical application. Kerdvibulvech et al. assessed the impacts of COVID-19 on the metaverse and digital games. Although this subject matter may not have been relevant to the paper, the methodology of assessing the impacts of COVID-19 could be useful if the paper needed to evaluate the impacts of a particular event. Lee et al. provided a strategic perspective on the application of the metaverse. Although this subject matter may not have been relevant to the paper, the methodology of providing a strategic perspective could be useful if the paper needed to provide a strategic perspective. Jamshidi et al. presented some AI-based methods for COVID-19 diagnosis and treatment using deep learning approaches. This methodology of using AI-based methods for COVID-19 could be relevant to the paper if it needed to present AI-based methods for a particular medical application. Further, de Moraes Rossetto et al. provided a framework based on blockchain for healthcare data privacy. Although this subject matter may not have been relevant to the paper, the methodology of providing a framework could be useful if the paper needed to provide a framework for a particular application.

In conclusion, the papers in the table had diverse subject matter and methodologies and can provide several potential connections to the paper, which could inform the methodology and approach undertaken in the research. The methodology and main findings of each paper were assessed for their relevance to the paper’s topic. Depending on the paper’s topic, certain methodologies could be useful for evaluating and presenting the topic. Table 2 provides information about notations used in the paper.

In the first section, which serves as the introduction to the study, we provide background information on the importance of assessing skeletal maturation in orthodontics and introduce the cervical vertebral maturation method. This method is commonly used by clinicians to determine the stage of skeletal maturation in growing individuals. Understanding the limitations of traditional approaches to this assessment, the authors propose a new deep-learning-based method to automatically detect and classify cervical vertebrae in radiographs, which is described in detail in the following section. Section 2 focuses on the CVM method, detailing cervical vertebral maturation stages and their clinical significance in assessing skeletal maturation in growing individuals. Section 3 presents the proposed CNN-based digital twin, a DL-based approach to automatically detect and classify cervical vertebrae in radiographs. The section provides a detailed description of the proposed method. Section 4 covers the results of the study, including the accuracy of the proposed algorithm in predicting cervical vertebral maturity stages. Section 5 provides a discussion of the study’s findings, including their implications and potential future directions for research in this area. Section 6 is the conclusion, summarizing the main contributions of the study and highlighting its potential clinical applications in orthodontics.

## 2. Cervical Vertebral Maturation Method

**Figure 1:**
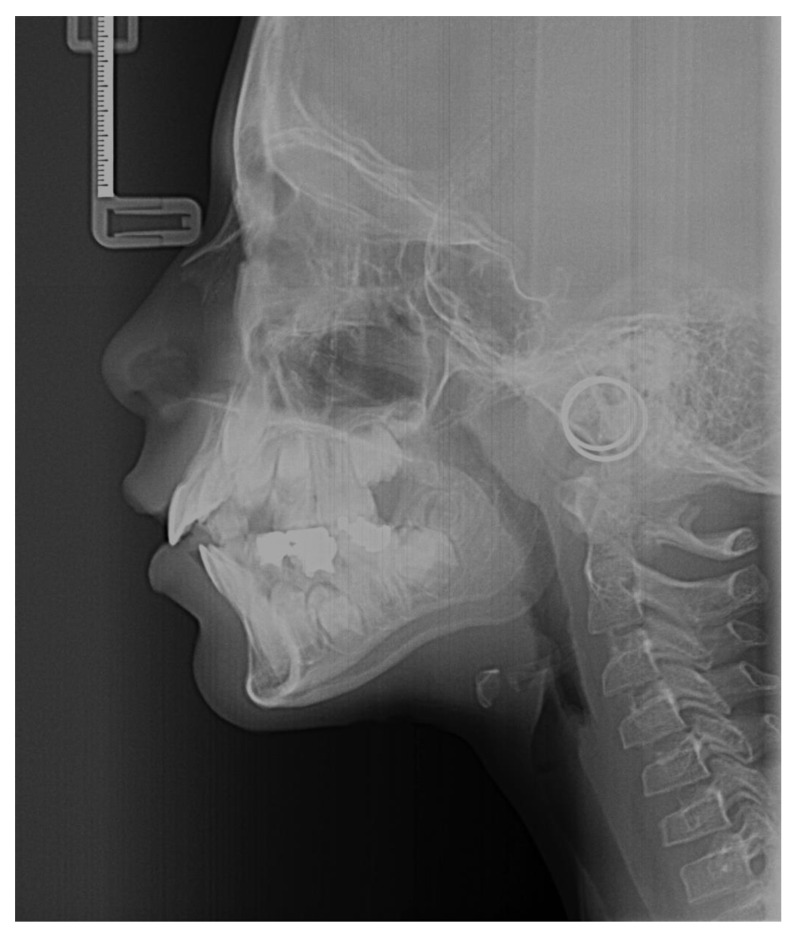
A sample of lateral cephalometric radiographs.

We have proposed to solve technical problems related to the assessment of skeletal maturity through CVM, specifically in orthodontic and orthopedic treatments. The current method of assessing skeletal maturity through lateral cephalometric radiographs has been subjective and can be inaccurate. Figure 1 depicts a lateral cephalometric radiograph. We have aimed to create a more reliable, efficient, and automated method for diagnosing CVM using advanced mathematical and deep learning algorithms. The proposed solution has involved creating a digital twin of CVM using a blockchain-based metaverse platform, which has allowed physicians, medical specialists, and researchers to access a variety of medical services in a virtual environment. The proposed DL method, using MobileNetV2, has provided an automated diagnosis process for upcoming CVM images, eliminating the need for additional sensors. We have aimed to develop a low-cost deep learning approach for diagnosis, anomaly detection, better design, and many more applications of the upcoming digital representations. The motivation for this research has been to address the limitations of the current methods of assessing skeletal maturity, which can be subjective, time-consuming, and expensive. The proposed solution has offered a practical and effective technique to benefit from the immersive characteristics of metaverse technology, creating virtual facilities and environments for patients, physicians, and researchers to access a variety of medical services.

The use of blockchain technology has enhanced reliability, safety, openness, and the ability to trace data exchange, bringing about cost savings through improved efficiencies. Additionally, the proposed solution has offered a low-cost alternative to traditional diagnosis and treatment methods, reducing the cost of diagnosis and treatment for patients. The appropriate timing for treatment is determined by identifying the maturity stage of the craniofacial skeleton and the growth phases favorable for structures such as the mandibular condyles and circum-maxillary sutures [14]. The assessment of skeletal maturity through CVM in lateral cephalometric radiographs is a substitute technique that eliminates the need for additional radiographs. This is because lateral cephalometric radiographs are already routinely taken for orthodontic diagnosis and treatment planning in orthodontic treatment.

**Figure 2:**
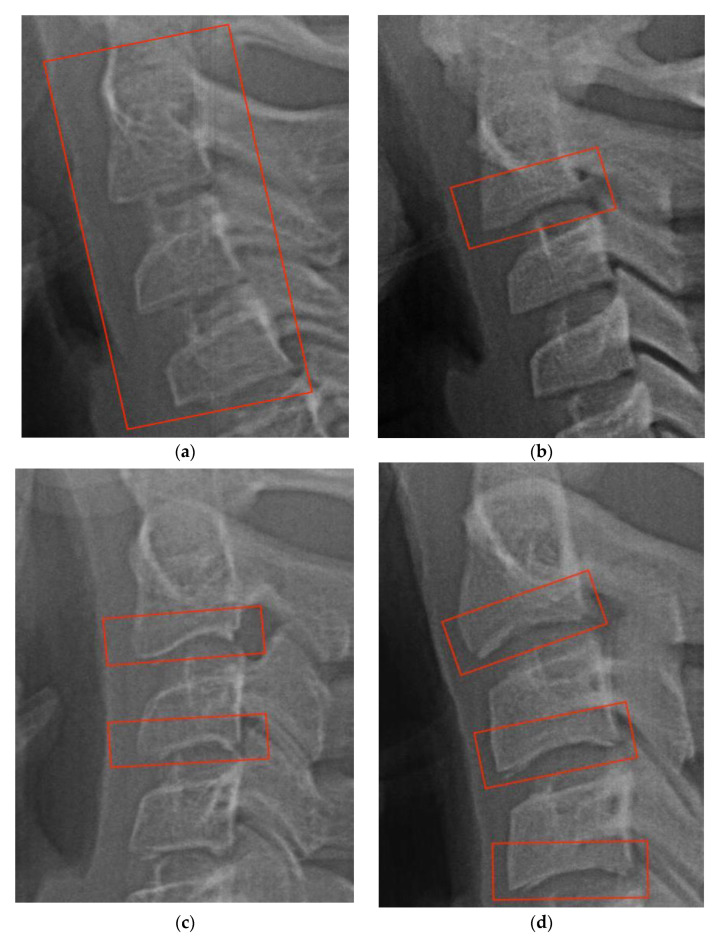
(**a**) The first stage. The inferior borders of the second, third, and fourth cervical vertebrae are flat and without indent; (**b**) the second stage. An indentation appeared on the inferior border of the second cervical vertebral, while the third and fourth ones are flat; (**c**) the third stage. A notch appears on the inferior border of the second and third cervical vertebrae; (**d**) the fourth stage. All three cervical bodies are indented.

**Figure 3:**
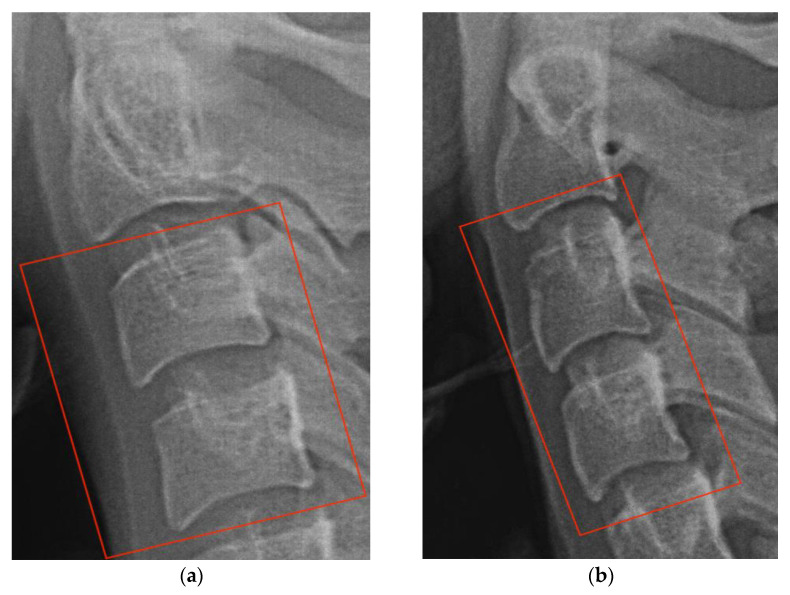
(**a**) The fifth stage. The bodies of the third and fourth cervical vertebrae are square in shape; (**b**) the sixth stage. The bodies of the third and fourth cervical vertebrae are rectangular in form.

The accuracy and consistency of CVM staging, as well as its correlation with the hand–wrist method, have been confirmed through numerous studies [15,16,17]. This method monitors the bodies of the second, third, and fourth cervical vertebrae. Two morphological attributes must be checked for deciding on the cervical stage. The first indicator is the presence or absence of an indentation on the inferior border of each of the three vertebral bodies. This indicator is used to distinguish between stages one to four. Figure 2 illustrates the variations in the first indicator and the corresponding stage. The second indicator, used to differentiate stages five and six, is the shape of the third and fourth cervical bodies. Figure 3 demonstrates the process of discriminating using the second indicator.

## 3. The Proposed CNN-Based Digital Twin within Blockchain

### 3.1. Motivation

The proposed digital twin focuses on improving the accuracy of determining the sixth stage of growth in patients using lateral cephalometric radiographs, which are X-ray images of the head. The sixth stage is considered a critical stage for various dental and medical applications and the most challenging stage to determine accurately. To address this challenge, the study proposes using deep transfer learning, a technique that leverages previous learning experiences to improve the results. The deep transfer learning approach uses X-ray images to determine whether the patient has reached their full growth potential. The study aims to create an accurate and reliable digital twin for determining the sixth stage, which will have important implications for various medical and dental treatments.

While digital twin-based systems require many sensors to detect changes in the target and the interaction with the target, this method does not need such infrastructures as it benefits from deep-learning-based computer vision as the measurement method. AI can deal with the enormous amount of sensor information from digital twins, which assists the various applications of modern society, ranging from wearable devices to smart buildings, but, in this study, we use a few X-ray images to train the network so that the digital twin can be updated without any extra sensors and hardware infrastructures. Currently, AI algorithms are used to recognize image objects, transcribe speech into text, match news items, predict user interests, and select relevant results from web searches. Recently, DL has been widely used in various fields as a new type of AI algorithm. DL is a representation learning method with multiple representation levels. It is obtained by combining simple but nonlinear relations, each of which transforms the representation of the original input level into a higher and more abstract level [18,19].

The neural network structure can learn complex functions through the combination of multiple transformations. For classification tasks, the higher-level representations enhance the important aspects of input data and help differentiate and remove irrelevant variations. This has resulted in significant advances in image and speech recognition using DL-based data analysis [19].

### 3.2. Metaverse, Blockchain, and Digital Twin

To develop a metaverse platform for the CVM parameter, certain key components are needed to establish a virtual space that is shared among users, such as doctors and patients, allowing them to engage with one another and digital resources. These components encompass several crucial elements [20,21,22,23,24,25]:

1. Decentralized identity: a system that provides doctors and patients with a unique digital identity that is self-sovereign and not controlled by any central authority. This allows users to participate in virtual activities while maintaining their privacy and security;
2. Virtual asset management: a system for creating, trading, and managing digital assets within the metaverse. This system should use blockchain technology to enable secure and transparent transactions;
3. World-building: a platform for creating and designing virtual environments where users can interact with each other and digital assets;
4. Interoperability: the ability for different metaverse platforms to communicate and interact with each other. This allows users to move their virtual assets and identities between different metaverse platforms;
5. Economy: a system for creating and managing a virtual economy within the metaverse. This includes virtual currencies, marketplaces, and other economic systems;
6. Governance: a system for managing the rules and regulations within the metaverse. This includes issues such as intellectual property, content moderation, and user behavior;
7. Security: a robust security system that protects users and their virtual assets from theft, fraud, and other cyber threats. This should include encryption, multi-factor authentication, and other security measures.

To ensure the privacy and security of users’ digital identities, a decentralized identity system should be used. This system uses blockchain technology and cryptographic algorithms to secure user information and prevent unauthorized access. This allows users to maintain control over their digital identities and protects them from identity theft and other cyber threats.

A metaverse platform should have a system for managing virtual assets, such as virtual currency, virtual real estate, virtual goods, and virtual services. This system should be based on smart contracts that enforce the rules of the metaverse and provide a secure and transparent way for users to trade and exchange virtual assets.

The platform should also have a system for creating and managing virtual worlds. This system should allow users to create and customize their own virtual environments and provide a framework for creating and managing virtual objects, animations, and interactive elements.

Interoperability between different virtual worlds is crucial, and the platform should provide a standard way for virtual objects and assets to move between virtual environments, as well as a standard way for users to interact with each other regardless of the virtual world they are in.

A metaverse platform should have a functioning economy that provides incentives for users to participate and contribute to the platform. This economy should include virtual currencies, virtual goods, and virtual services that can be bought, sold, and traded within the metaverse.

The platform should have a governance system that allows users to make decisions and enforce rules and regulations. This system should be transparent and fair and allow for the creation of decentralized autonomous organizations (DAOs) to represent user interests in the metaverse.

Security is a critical issue, and the platform should use cryptographic algorithms to secure user information and virtual assets. It should have a robust system for detecting and mitigating malicious activities.

In addition to these key elements, a metaverse platform can also incorporate digital twins to enhance its capabilities. Digital twins are digital representations of physical objects or systems, providing a way to monitor, control, and optimize their performance in real time. In the context of the metaverse, digital twins can be used to bring physical objects and systems into the virtual world, providing a more immersive and interactive experience for users.

**Figure 4:**
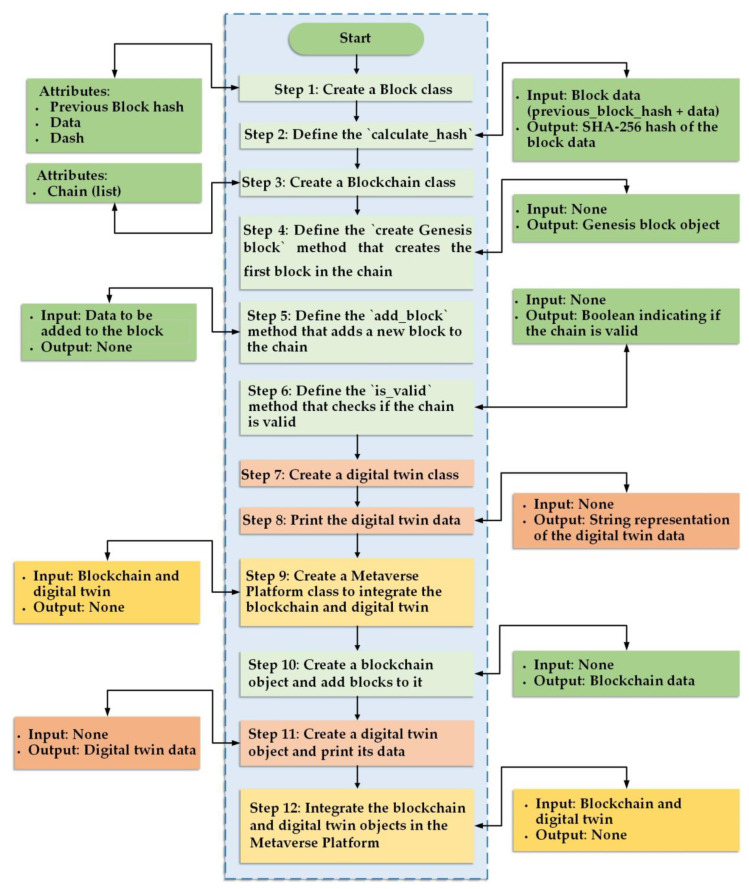
An illustration of a basic blockchain platform implementation for the system.

Figure 4 illustrates the implementation of a very simple blockchain platform for the system. In a blockchain, a “block” refers to a unit of data containing a set of transactions recorded on the blockchain. The “Create a Block” class is a programming concept that represents a blueprint for creating a new block on the blockchain. It defines the properties and methods necessary for creating a new block, such as the block’s unique identifier, the transactions it contains, and the hash value used to link it to the previous block in the chain.

“Calculate_hash” is a method used in blockchain technology to generate a unique digital signature for each block in the blockchain. The hash value is a fixed-length string of characters that is computed using complex mathematical algorithms, and it serves as a digital fingerprint for the block’s data. The hash value is unique to each block and changes if any data in the block are modified, ensuring the blockchain’s security and immutability. To calculate the hash value of a block, the method takes the data that are stored in the block, including the transactions, timestamp, and previous block’s hash value, and runs them through a hash function, such as SHA-256. The resulting hash value is then added to the block as its unique identifier and is also used to link the block to the previous block in the blockchain. By using the “Calculate_hash” method, the integrity of the data on the blockchain can be maintained, and any attempts to modify or tamper with the data can be easily detected.

“Create a Blockchain” class is a programming concept used to represent the blueprint for creating a new blockchain in blockchain technology. It defines the properties and methods that are necessary for creating and managing a blockchain, including adding new blocks, validating the chain, and resolving conflicts. The class consists of a chain of blocks, where each block is linked to the previous block using its unique hash value. Each block contains a set of transactions and a timestamp, and the entire chain is secured using complex cryptographic algorithms that ensure its immutability and security. The “Create a Blockchain” class also includes methods for validating the chain by checking the integrity of each block and its links to the previous block. If any inconsistencies or tampering are detected, the blockchain is rejected, and the validation process is restarted. Another important method included in the “Create a Blockchain” class is resolving conflicts. When two or more nodes create a block at the same time, a conflict arises, and the chain needs to be updated to include the correct block. This method defines the process for resolving conflicts, ensuring that the chain remains consistent across all nodes on the network. By using the “Create a Blockchain” class, developers can efficiently create and manage a new blockchain and ensure the integrity and security of the data stored on the chain.

“Create_genesis_block” is a method used in blockchain technology to create the first block in a new blockchain. This block is also known as the “genesis block” and serves as the starting point of the chain. The “Create_genesis_block” method is typically called when a new blockchain is created, and it creates a block with a predefined set of data and hash value. The data stored in the genesis block typically include information such as the timestamp of the block’s creation, the initial set of users or stakeholders, and any other relevant information about the blockchain. The hash value of the genesis block is typically a hardcoded value or generated using a predefined hash function. Once the genesis block is created, it is added to the chain as the first block, and all subsequent blocks are linked to it using its hash value.

The genesis block cannot be modified or removed from the chain and serves as the starting point for all transactions and blocks that are added to the chain. By using the “Create_genesis_block” method, developers can efficiently create the initial block of a new blockchain, ensuring that the chain starts with a predefined set of data and hash value and that subsequent blocks can be linked to it correctly.

“Add_block” is a method used in blockchain technology to add a new block to the chain. The method takes as input the data for the new block, including the transactions and any relevant metadata, and generates a unique hash value for the block. The hash value is calculated using a hash function, such as SHA-256, and is based on the data stored in the block as well as the hash value of the previous block in the chain. Once the hash value for the new block is calculated, the block is added to the chain, and its hash value is used to link it to the previous block in the chain. This linking process ensures the security and immutability of the blockchain as any attempts to modify the data in a block will result in a change in its hash value, which will be immediately detected by the network. The “Add_block” method also includes error-checking and validation procedures to ensure that the data stored in the new block are valid and conform to the rules and protocols of the blockchain. If any errors or inconsistencies are detected, the block is rejected, and the validation process is restarted.

“Define the __str__ method for the Digital twin class to print the digital twin data” is a task or instruction that asks a programmer to implement a special method called “str” for the digital twin class in their code. The “str” method is used to define the string representation of an object, and, in this case, it is used to print the data of the digital twin object in a readable format. When the programmer defines the “str” method for the digital twin class, they can specify the information they want to print about the digital twin, such as its unique ID, properties, and relationships to other objects in the system. Once the “str” method is defined for the digital twin class, the programmer can call it to print the string representation of the digital twin object in a human-readable format. This can be useful for testing and debugging purposes as it allows the programmer to quickly inspect the properties and relationships of the digital twin without having to examine the object directly in the code. Overall, defining the “str” method for the digital twin class is a common programming task that can help make the code more readable and easier to understand, and can help the programmer interact more easily with and manage the digital twins in their system.

“Create a Metaverse Platform class to integrate the Blockchain and Digital twin classes” is an instruction that asks a programmer to create a new class in their code, called the metaverse platform class, which will integrate the blockchain and digital twin classes. This integration aims to create a platform that allows digital twins to be securely stored and managed on the blockchain, ensuring that they are tamper-proof and immutable. The metaverse platform class will take the blockchain and digital twin classes as input and will output nothing. By integrating the blockchain and digital twin classes, the metaverse platform class can enable developers to easily create and manage digital twins, as well as interact with them through a secure, decentralized platform. The metaverse platform class can also help to ensure the consistency and accuracy of the data stored on the blockchain and can make it easier to analyze and understand the relationships and dependencies between different digital twins. Overall, creating the metaverse platform class is a critical step in developing a metaverse, a virtual world that is created by combining elements of the physical and digital worlds. By integrating the blockchain and digital twin classes, the metaverse platform class can provide a secure, decentralized platform for managing and interacting with digital twins, which can help to unlock new possibilities for innovation and collaboration in a wide range of industries and applications.

The three steps described here are part of a larger process of creating a metaverse platform. A metaverse is a virtual world that combines elements of the physical and digital worlds, allowing users to interact with each other and with digital objects in a shared environment.

Step 10 involves creating a blockchain object, which is a data structure used in decentralized systems to store a series of blocks containing transaction records. A blockchain is essentially a ledger that can be used to record any type of transaction, such as the transfer of cryptocurrency, ownership of a digital asset, or the execution of a smart contract. Each block in the chain contains a unique cryptographic hash, a timestamp, and a record of transactions. When a new block is added to the chain, it is verified by other nodes in the network to ensure its integrity.

To implement this step, one would need to use a programming language or a platform that supports blockchain development, such as Ethereum, Hyperledger Fabric, or Corda. One would create an instance of a blockchain object and then add blocks to it to record transactions, either by calling functions or using APIs provided by the platform.

Step 11 involves creating a digital twin object, which is a virtual representation of a physical object, system, or process. A digital twin can be used to monitor and control physical systems in real time, simulate changes and optimize performance, or enable remote maintenance and repair. A digital twin typically consists of three components: a virtual model of the physical system, a data infrastructure to collect and analyze sensor data, and a set of analytics tools to generate insights and recommendations.

To implement this step, one would need to develop a digital twin using modeling and simulation software or a platform that supports digital twin development, such as Siemens MindSphere, Microsoft Azure Digital twins, or IBM Watson IoT. One would create an instance of a digital twin object and populate it with data, such as the geometry and material properties of the physical object, the location and orientation of sensors, and the data processing algorithms used to analyze the data.

Step 12 involves integrating the blockchain and digital twin objects in the metaverse platform, which is the ultimate goal of the process. Integration means making the blockchain and digital twin objects work together seamlessly to enable new applications and use cases. For example, a blockchain could be used to store the ownership of a digital twin, the history of changes made to it, and the results of simulations run on it. A digital twin could be used to collect sensor data from a physical system, generate alerts based on predefined rules, and trigger transactions on the blockchain.

To implement this step, one would need to design and develop a set of APIs and protocols that enable the blockchain and digital twin objects to communicate with each other. This could involve defining a common data model, creating a set of RESTful APIs, or implementing a set of smart contracts on the blockchain. Once the integration is complete, one could deploy the metaverse platform and start building new applications and services that leverage the power of blockchain and digital twin technologies.

### 3.3. Implementation of the Platform

Table 3 shows the pseudocode of implementation of the platform. The pseudocode suggests building a chain to store each patient’s information. The “patient_chain_class” is provided to demonstrate one possible implementation of the chain belonging to a patient. The class contains five main functions:

“initialize”: This function is called every time a new chain is created. It defines the class’s attributes and creates an empty block called the genesis block, the first block of the chain. Since there is no previous block at the beginning, to build the genesis block, the previous block’s hash is set to an arbitrary number of 1.

“new_block”: This function is called every time a new block is created. Everything that needs to be stored in a block is defined in this function:

“index”: the block number

“image”: the latest cephalometric radiograph is hashed and stored in the current block.

“previous_hash”: the previous block is hashed and stored in the current block. This information aims to link each block to the previous one and create the desired chain-like structure.

“prediction”: the output of the proposed MobileNetV2 algorithm on the latest cephalometric radiograph is stored in this section. To receive this value, the latest cephalometric radiograph would be sent to a cloud-stored program defined in the pseudocode as the “run_classification_algorithm”. This function will be explained more later in this section. Finally, the output of the mentioned function would be sent back to the client and stored in this section.

Note that, for simplicity, it is assumed that each block contains one cephalometric radiograph and its corresponding diagnosis result provided by the deep learning algorithm. Of course, it goes without saying that any other information related to the patient and their medical history can be stored in each block. In addition, with sufficient memory and time complexity analysis, one can store several images in each block.

Eventually, the block will be added to the chain, and the attributes of the chain class will be reset to the default values.

“new_image”: This function receives the latest cephalometric radiograph and stores its hash and raw 2D array to the attributes “current_image_hash” and “current_image”, respectively.

“hash”: This function receives the content of the previous block and calculates its hash after turning it into a JSON format. It is used in building the new block.

“new_prediction”: This function sends the current cephalometric radiograph to the “run_classification_algorithm”, hosted on a cloud. The response value is hashed and returned.

The deep-learning-based classification algorithm is assumed to be running and hosted on a cloud-based platform. The pseudocode has illustrated the prediction procedure using three functions:

“build_model”: This function loads MobileNetV2 architecture and replaces the last softmax layer with a sigmoid layer. We propose adding a dropout layer before the sigmoid layer with a dropout probability of 40%. The function then returns the task-modified version of MobileNetV2 architecture.

“load_model_checkpoint”: A model’s checkpoint contains a history of the model throughout the training process and the final trained weights. To demonstrate more, you can find the checkpoints of all five models, which have been trained during the 5-fold cross-validation process described in the paper in the following link:

https://drive.google.com/drive/folders/1WrGLst8ASPtrBCg5pL8n87tTWY60gMfx?usp=sharing (accessed on 17 April 2023).

This function loads the checkpoint of the desired model and returns it. Again, checkpoints are assumed to have been stored on the same cloud-based platform.

“run_classification_algorithm”: This function defines the prediction process. It is part of the program that receives a request from the blockchain client and returns the prediction result. First, the desired MobileNetV2 architecture is loaded through the function “build_model”. Then, the selected checkpoint is loaded by calling the function “load_model_checkpoint”. With the base architecture and the trained weights, the default weights are replaced with the trained weights stored in the loaded checkpoint. Before starting the prediction process, the model’s mode is set to evaluation to deactivate the dropout layers during prediction. The next step involves the preprocessing of the image, where three copies of the only channel of the grayscale cephalometric radiograph are generated and stacked together to replicate the RGB form expected by the MobileNetV2. Afterward, the image is resized to have the desired shape, and, lastly, the pixel values are normalized. Finally, the image is run through the model, and the prediction is outputted.

### 3.4. MobileNetV2

MobileNetV2 introduced a new convolutional neural network architecture designed specifically for mobile and resource-constrained environments. It aimed to push the state of art for mobile computer vision models by reducing the number of operations and memory required while retaining the same level of accuracy. The main contribution of the method was the introduction of a novel layer module called the inverted residual with a linear bottleneck. This module takes a low-dimensional compressed representation as input, expands it to a high dimension with a lightweight depthwise convolution, filters the features, and projects them back to a low-dimensional representation using a linear convolution. The official implementation of the architecture was made available as part of the TensorFlow-Slim model library [26].

In other words, MobileNetV2 is a new mobile architecture that aims to improve the performance of mobile models in various tasks and benchmarks. It uses an inverted residual structure where the connections are between thin bottleneck layers and lightweight depthwise convolutions in the intermediate expansion layer to filter features [26]. The design of MobileNetV2 focuses on removing non-linearities in the narrow layers to maintain representational power and provides a flexible framework for further analysis. It has been tested on ImageNet classification, object detection, and image segmentation, and its trade-offs between accuracy, operations, latency, and number of parameters have been evaluated. The authors also present ways to efficiently use MobileNetV2 for object detection in a framework named SSDLite and for building mobile semantic segmentation models using Mobile DeepLabv3 [26].

### 3.5. Dataset

The dataset consists of 319 lateral cephalometric radiographs, which are X-ray images of the head used for orthodontic purposes. An experienced orthodontist has classified each radiograph into one of six stages using the CVM (cervical vertebral maturation) method. The data are then divided into two groups: the positive class and the negative class. The positive class includes all the radiographs belonging to the sixth stage, while the negative class includes all the radiographs belonging to stages one through five. There are a total of 32 greyscale images from each of the five stages in the negative class, making a total of 160 greyscale images in this class. On the other hand, the positive class contains 159 greyscale images of the sixth stage.

## 4. Results

A deep transfer learning approach has been used to classify the images into two classes. MobileNetV2 architecture with the pre-trained weights on the ImageNet dataset has been picked as the base model [26]. To adapt the architecture to the binary classification task, the last layer was replaced with a sigmoid layer to output the probability of reaching the sixth stage. Before the sigmoid layer, a dropout layer with a dropout probability of 0.4 was added.

No image processing technique was used. To adapt the input images to the expected input format by the pre-trained MobileNetV2, the images were resized to 224 × 224 × 3. Since the pre-trained MobileNetV2 desires RGB images with three color channels as the input, the only channel of greyscale images was repeated three times to satisfy the desired three channels. The pixel values were normalized with a mean of 0.485, 0.456, and 0.406 and a standard deviation of 0.229, 0.224, and 0.225 for each channel, respectively.

The training part was implemented using Python 3 and PyTorch 1.13. The code was written and run in the Google Colab environment with an A100-SXM4–40 GB Nvidia GPU accelerator. The log-loss (binary cross-entropy loss) was used, which is defined in Equation (1).

(1) $$\log\_loss(y,\hat{y})=-y\log\hat{y}-(1-y)\log(1-\hat{y})$$

In Equation (1), y is the true label 0 or 1, where 1 represents reaching the sixth stage. $\hat{y}$ is the probability of belonging to the positive class (y = 1), provided by the last sigmoid layer. An Adam optimizer was used with a learning rate of $10^{-4}$ and a $\beta_{1}$ of 0.85 and a $\beta_{2}$ of 0.995. To prevent overfitting, an L2 penalty (weight decay) of $10^{-4}$ was set.

To further reduce the chance of overfitting, two data augmentation methods were utilized using predefined transforms in the torchvision library: performed random auto contrast with a probability of 0.6 and randomly adjusted sharpness with a sharpness factor of 2 and a probability of 0.5. In order to obtain a better estimation of the model’s performance, a 5-fold cross-validation with a batch size of 20 was used. The number of epochs for each fold was set to 8.

In this section, we describe the structure of the study and the different types of data analysis that were conducted. The evaluation was completed using a technique called 5-fold cross-validation to evaluate the performance of the algorithm. This technique involves dividing the dataset into five folds and training a model, from scratch, on four folds while using the remaining fold for validation. Each fold would contain $\frac{N}{5}$ examples, where N is the total number of samples. More specifically, five models were trained, and each of them was evaluated on a different fold. This way, the algorithm was tested on all the examples. Four different metrics were calculated to measure various aspects of each model on their evaluation fold: precision, recall, accuracy, and the total loss (cost) [27].

As we discussed before, the proposed model outputs the probability of belonging to the positive class. On the other hand, a desired prediction output is 0 or 1. Therefore, to convert probabilities to predictions, a threshold of 0.5 was used in the calculation of discussed metrics. Consequently, a probability of 0.5 or higher was regarded as a prediction of 1 (sixth stage).

Precision aims to measure the reliability of the model in predicting the positive class, in other words, how reliable it is when the algorithm recognizes a radiograph as the sixth stage. Precision is calculated using the formula provided by Equation (2).

(2) $$Precision=\frac{\sum_{i=1}^{N}1\{{\hat{y}}_{i}\geq threshold\wedge y_{i}=1\}}{\sum_{i=1}^{N}1\{{\hat{y}}_{i}\geq threshold\}}$$

As is clear in Equation (2), precision provides the fraction of examples that the algorithm correctly classified as the sixth stage, considering the total number of samples that the algorithm classified as the sixth stage. It means that, as the number of false positives increases, the precision decreases. Diagnosing a patient who has not reached the sixth stage as a positive example can affect their dental and medical procedure adversely. Therefore, precision is an essential metric to be examined.

Recall shows the sensitivity of the algorithm. It indicates the chance of recognizing a positive sample by the algorithm. Equation (3) provides the formula for calculating recall.

(3) $$Recall=\frac{\sum_{i=1}^{N}1\{{\hat{y}}_{i}\geq threshold\wedge y_{i}=1\}}{\sum_{i=1}^{N}1\{y_{i}=1\}}$$

As demonstrated in Equation (3), recall measures the fraction of radiographs belonging to the sixth stage that the algorithm was able to correctly recognize. Hence, as the number of false negatives increases, the recall decreases. While false negatives are less harmful, measuring recall is important to make sure the algorithm is not biased toward negative decisions [27,28].

Since the dataset is balanced and both classes contain a similar number of samples, accuracy can be used to measure how accurate the models are in identifying the true class. It can be calculated using Equation (4).

(4) $$Accuracy=\frac{\sum_{i=1}^{N}1\left\{{\hat{y}}_{i}\geq threshold\wedge y_{i}=1\right\}+\sum_{i=1}^{N}1\{{\hat{y}}_{i}<threshold\wedge y_{i}=0\}}{\sum_{i=1}^{N}1}$$

Total loss (cost) shows the success of the optimization algorithm. This is the actual metric that is minimized during the training process; therefore, a lower total loss (cost) indicates a better job in optimizing the learning objective and finding a sufficient local minimum. This metric helps to compare the learning of different models and recognize overfitting by examining the gap between the value of the metric on the training and evaluation set. Total loss (cost) on N samples is defined as follows:

(5) $$total\;loss\left(cost\right)=\frac{1}{N}\sum_{i=1}^{N}\log\_loss(y_{i},{\hat{y}}_{i})$$

**Figure 5:**
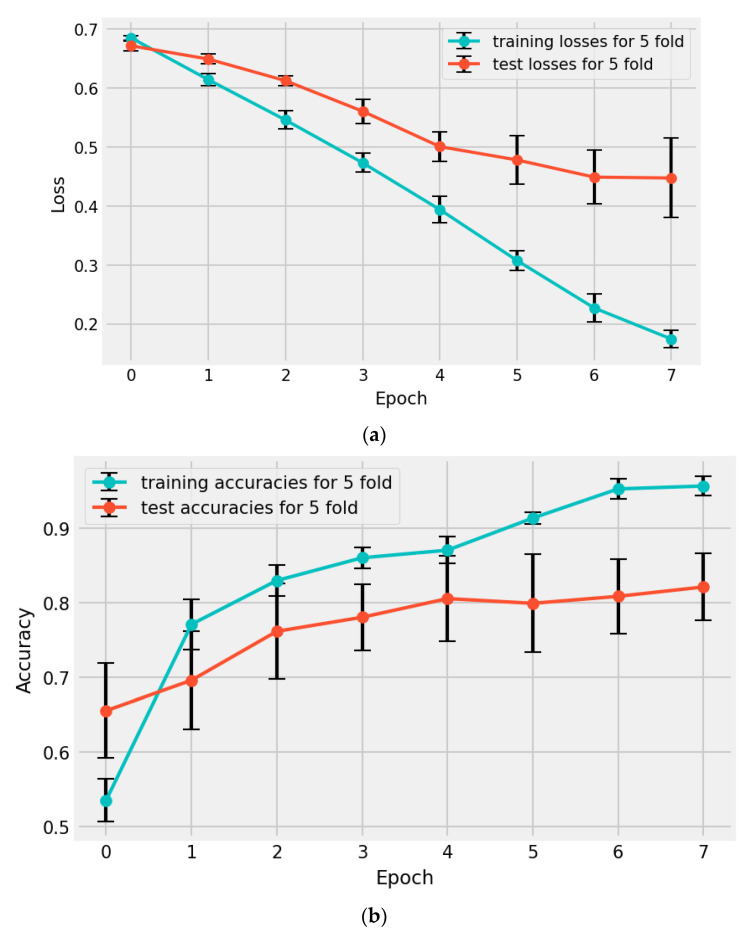
(**a**) Mean and standard deviation of all five folds’ losses per epoch; (**b**) mean and standard deviation of all five folds’ accuracies per epoch.

**Figure 6:**
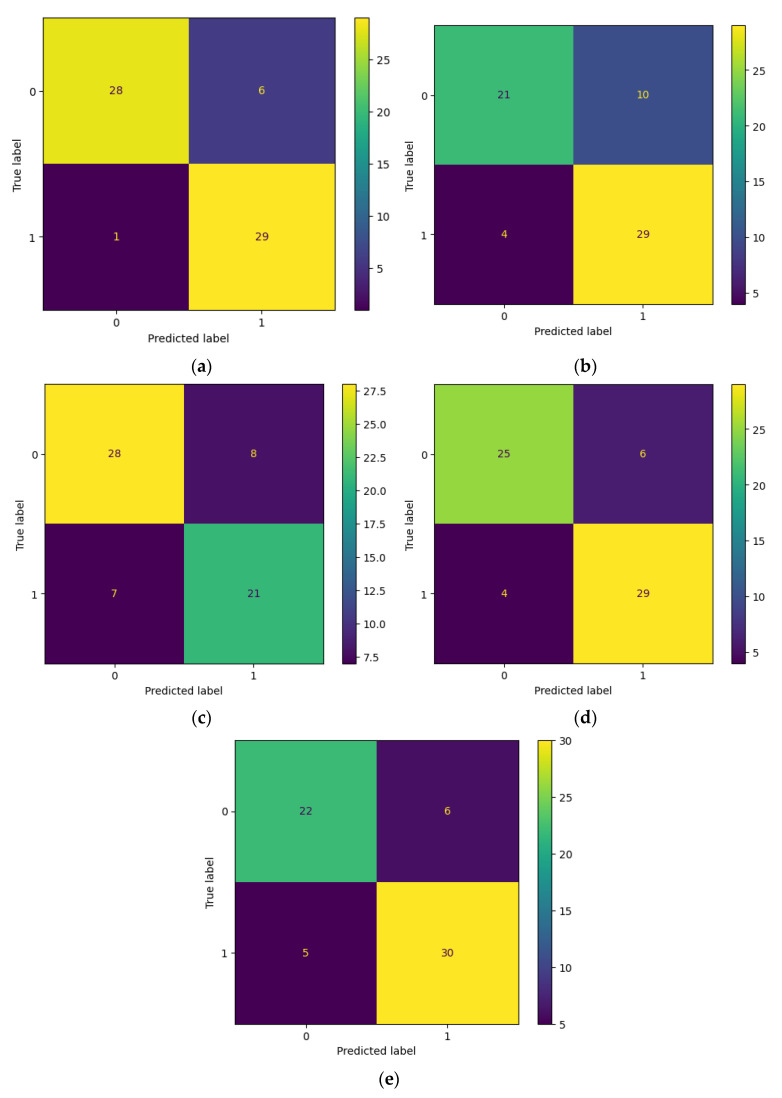
The confusion matrix for the validation sets of the (**a**) first, (**b**) second, (**c**) third, (**d**) fourth, and (**e**) fifth folds show that the number of false positives is greater than false negatives in all cases. Despite this, the consistency of false and true values suggests that the model is effectively identifying indicators for the sixth stage. It can be concluded that, with more data points, the number of false positives could be reduced significantly given that the only variable between each fold is the training and validation set.

Finally, the mean and standard deviation of all models’ performances on each metric were calculated. Using this evaluation approach on the available small dataset provided a more reliable measurement of the algorithm and its uncertainty in real-world scenarios. Table 4 displays the resulting discussed metrics for each model on its evaluation fold. To examine the progress of the models during the training, Figure 5 illustrates the mean and standard deviation of total loss and accuracy of the models during each epoch. The figure visually represents how the overall performance of models improved over time as they were trained for more epochs. Figure 6 presents the confusion matrix for each fold, a table that summarizes the number of true positives, true negatives, false positives, and false negatives in the model’s predictions. This type of analysis allows for a detailed examination of the model’s accuracy and can help identify areas for improvement.

To examine the effect of the selected threshold, we evaluated the models using receiver operating characteristic (ROC) curve. The ROC curve represents the value of the true positive rate (recall) and false positive rate for all the different choices of threshold values from 1 to 0. A perfect split would represent a curve with an area under curve (AUC) equal to 1. True positive rates (recalls) are calculated using Equation (3), and false positive rates are calculated using Equation (6).

(6) $$False\;Positive\;Rate=\frac{\sum_{i=1}^{N}1\{{\hat{y}}_{i}\geq threshold\wedge y_{i}=0\}}{\sum_{i=1}^{N}1\{y_{i}=0\}}$$

**Figure 7:**
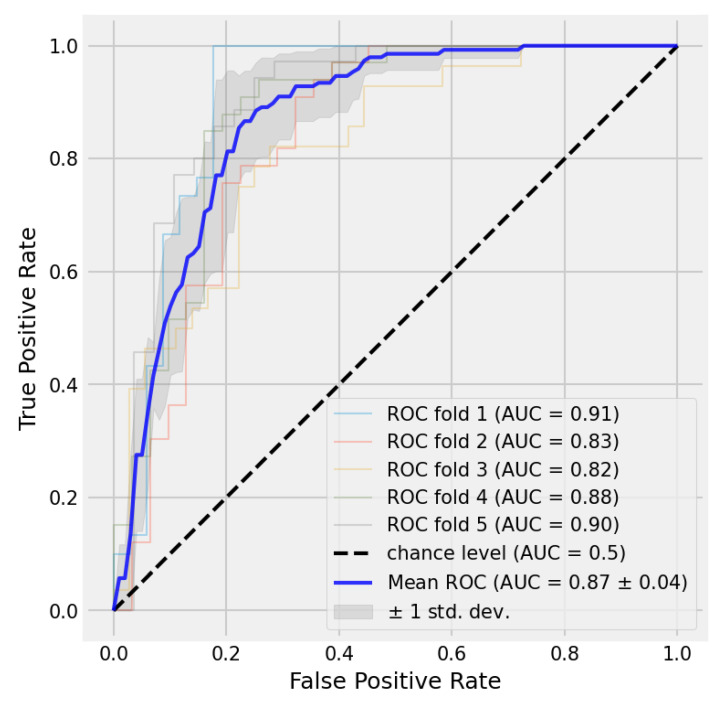
Receiver operating characteristic (ROC) curve for each fold. The mean ROC is shown in blue color.

The resulting curve for each fold is depicted in Figure 7. The area under curve (AUC) for mean ROC is 0.87±0.04. Not only is it a good area but the curve also indicates a reduction in the number of false positives by choosing a threshold larger than 0.5.

## 5. Discussion

As society moves toward digitalization, it is essential for researchers to examine the challenges of digitalization in different fields. Digitalization of the different aspects of healthcare is of particular importance since its applications can hugely affect people’s lives around the world. More importantly, an accurate diagnosis is the most crucial facet of healthcare applications. Not only does automating the diagnosis process increase the accessibility of reliable and equal treatment for everyone around the world but it also leads to higher-quality examinations of patients. This work demonstrated the potential of deep-learning-based computer vision approaches in building digital twins for different diagnosis procedures by examining an essential process in dental and jaw treatment. In the article, we propose a practical and effective technique for creating digital twins of dental issues in the Metaverse using advanced mathematical and deep learning algorithms. Our proposed technique is simple, fast, and suitable for physicians and medical specialists, and it does not require additional sensors. We also demonstrate the applicability and suitability of the introduced approach by designing and implementing a comprehensive conceptual framework for creating digital twins of CVM based on MobileNetV2 within a blockchain ecosystem. The high performance of our proposed model on a small dataset demonstrates that low-cost deep learning can be used for diagnosis, anomaly detection, better design, and many more applications of the upcoming digital representations. Finally, we show how digital twins can be developed for dental issues with the lowest hardware infrastructures, reducing the costs of diagnosis and treatment for patients.

We showed that, with the emergence of cost-efficient deep models, we could build accurate digital twins for diagnosis purposes. In addition, due to the efficiency of these models, they can be used in embedded systems at radiology centers or medical clinics. On top of that, these models are faster to train, which paves the way for online training of the models in production on different platforms, such as Metaverse. Online training of the models during production is a vital research path since it can provide the model with more diverse datasets, which leads to a wide range of cases. As a result, the quality of treatment will increase due to the richer knowledge of the model compared to a limited geographic or institutional experience.

We also demonstrated the effectiveness of transferring previous knowledge to enhance the accuracy of the models. While we had to use pre-trained weights on the ImageNet dataset because of the limited access to medical data, building digital twins and connecting institutions and experts through platforms such as Metaverse can also address this issue. Incorporating models in the Metaverse and IoT systems, and constantly training them with medical images, will provide the healthcare community with models whose weights are specialized in interpreting medical images. As a result, it will revolutionize the research of multi-task learning and transfer learning and provide the community with more accurate models. Another notable consequence of having these expert models is having great teacher models used in knowledge distillation methods. As a result, more memory-efficient models can also be trained with a minor decrease in accuracy, as well as providing us with more options to develop various unsupervised approaches. These memory-efficient student models can then be used in the Internet of Medical Things (IoMT).

The applications of digital twins will not be confined to diagnosis. Minor tweaks in the classifiers can assist in building more realistic medical digital twins by using their knowledge of the physical world to detect anomalies in digital assets. Therefore, educational institutions will be equipped with indistinguishable digital counterparts of medical assets for educational purposes. From a performance point of view, our model showed a relatively good balance between the false positive and false negative rates, with a higher false positive in most experiments. Since a lower false positive rate is preferred in medical applications, the problem can be addressed by deploying the model in Metaverse or IoT systems and online training of the model with more positive samples. In addition, the model can always be accompanied by the experts’ diagnosis or used in an ensemble setting with other models.

## 6. Conclusions

In recent years, there has been a growing interest in the application of the Metaverse, AI, and digital twins in healthcare. However, the implementation of these technologies in healthcare systems has faced certain limitations, particularly with regard to the digitalization of medical disorders and assets, such as digital twinning. The complexity of medical disorders has made it difficult to create digital twins that are scalable, flexible, and usable in healthcare systems. To address this issue, we propose a digital twin of CVM, a critical factor in certain dental treatments and surgeries, which can accurately discriminate its stages with the highest rate of accuracy and lowest cost. Our approach involves the implementation of a comprehensive framework for recognizing CVM within a digital twin that is based on blockchain and has been implemented in a blockchain-based Metaverse. We utilized MobileNetV2, a mobile architecture that improves the performance of mobile models in various tasks and benchmarks, as the engine for our proposed digital twin. The proposed digital twinning technique is simple, fast, and suitable for physicians and medical specialists, and it is adaptable to the Internet of Medical Things due to its low latency and computing costs. We used deep-learning-based computer vision as a measurement method, which does not require additional sensors, making a significant contribution to the digital twin method. Our findings demonstrate that digital representations are practical and trustworthy in tracking and identifying issues, and the proposed platform can accurately discriminate between the sixth stage and other stages of CVM.

There are several potential avenues of exploration. There could be further investigation into the creation of digital twins of other dental issues within the Metaverse, and whether this approach can be applied to other medical domains. In addition, exploring the use of blockchain-based Metaverse platforms in creating more accurate and efficient dental treatments and surgeries may be worthwhile. Furthermore, improving the scalability and flexibility of the proposed approach could increase its potential for wider use in healthcare systems. It would also be interesting to delve into the immersive interaction between doctors and patients using digital twins and Metaverse platforms as this could have a significant impact on the efficiency of the healthcare system.

## Figures and Tables

**Table 1:** An overview of the studies that are relevant to the proposed approach.

Author(s)	Journals	Subject	Methodology or Equipment	Main Findings
Korde, S.J et al. [1]	Int. J. Sci. Res	Skeletal Maturity Indicators	Reviewing several methods	Assessing and categorizing some conventional methods to measure skeletal maturity
Rolland-Cachera et al. [2]	The American journal of clinical nutrition	Assessment of growth	Assessment of growth	Evolution of growth based on references and growth parameters
Fang, D. et al. [3]	Journal of Oral and Maxillofacial Surgery	Treatment perspective	Clinical application of concentrated growth factor	A hybrid approach to repair jaw defects based on growth factor
Ferrillo, M. et al. [4]	Journal of back and musculoskeletal rehabilitation	Assessment of growth	A systematic review	Determining the reliability of cervical vertebral maturation
Hamet, P. et al. [5]	Metabolism	AI techniques for medicine	Assessment of several methods	Recommending different AI methods for different medical issues
Jamshidi, M.B et al. [6]	ICCKE conference	AI techniques for complex problem	A hybrid echo state network	AI approaches for hypercomplex pattern recognition, classification, and big data analysis
Jamshidi, M.B. et al. [7]	Mechatronika conference	AI techniques for medicine	Three ML methods	Creating cancer digital twins
Kerdvibulvech, C. et al. [8]	HCII conference	Human–Computer Interaction	Survey	Assessing impacts of COVID-19 on metaverse and digital games
Lee, C.W. et al. [9]	International Journal of Environmental Research and Public Health	Application of the metaverse	Assessment and suggestion	Providing a strategic perspective
Jamshidi, M. et al. [10]	IEEE Access	Artificial intelligence and COVID-19	Deep learning approaches	Presenting some AI-based methods for diagnosis and treatment
de Moraes Rossetto et al. [13]	Sensors	Data privacy in healthcare	Blockchain approach	Providing a framework based on blockchain for healthcare data privacy

**Table 2:** Mathematical symbols and abbreviations.

Symbol	Definition
$1\left\{statement\right\}$	Denotes the “indicator function”. The indicator of a true statement is equal to 1 and the indicator of a false statement is equal to 0.
∧	Denotes the “logical and”. X ∧Y is true if both statements, X and Y, are true. Otherwise, it is false.
=	Denotes “equality”. X =Y is true if the values of X and Y are equal.
≥	Denotes “greater than or equal to”. X ≥Y is true if the value of X is greater than or equal to the value of Y.
<	Denotes “less than”. X <Y is true if the value of X is less than the value of Y.
$\sum_{i=1}^{N}$	Denotes the sum of N terms, where N is the number of samples.
${\hat{y}}_{i}$	The predicted probability of belonging to the positive class. AI model receives the ith sample and outputs this probability.
$y_{i}$	The true label of the ith sample. It is 0 or 1, where 1 means the radiograph belongs to the sixth stage of growth.

**Table 3:** Pseudocode of implementation of the platform within blockchain.

Pseudocodes of Implementation
DEFINE CLASS patient_chain_class():
DEFINE initialize FUNCTION():
SET chain TO AN EMPTY LIST
SET curret_image_hash TO AN EMPTY STRING
SET current_image TO AN EMPTY 2D ARRAY
new_block(previous_hash = 1)
DEFINE new_block FUNCTION(previous_hash = False):
SET block TO {
‘index’: LENGTH OF chain + 1,
‘image’: current_image_hash,
‘previous_hash’: previous_hash == 1 ? 1: hash(last_block()),
‘prediction’: previous_hash == 1 ? ‘No Prediction (First Block)’: new_prediction()
}
SET current_image_hash TO AN EMPTY STRING
SET current_image TO AN EMPTY 2D ARRAY
ADD block TO chain
RETURN block
DEFINE new_image FUNCTION(image):
SET current_image_hash TO HASH OF image
SET current_image TO image
DEFINE new_prediction FUNCTION():
SET prediction TO run_classification_algorithm(current_image)
RETURN HASH OF prediction
DEFINE hash FUNCTION(block):
SET block_string TO JSON FORMAT OF block
RETURN HASH OF block_string
DEFINE last_block FUNCTION():
RETURN LAST ELEMENT OF chain
SET patient_chain TO AN INSTANCE OF patient_chain_class()
SET img TO IMAGE OF PATIENT
new_image(img)
new_block()
DEFINE build_model FUNCTION():
SET model TO mobilenet_v2
REPLACE THE LAST CLASSIFIER WITH sequential(
dropout_layer(*p* = 0.4),
linear_layer(in_features = 1280, out_features = 1, bias = True),
sigmoid_activation
)
RETURN model
DEFINE load_model_checkpoint FUNCTION(model_name):
RETURN CHECKPOINT OF model_name
DEFINE run_classification_algorithm FUNCTION(image):
SET model TO build_model()
SET checkpoint TO load_model_checkpoint(model_name)
SET THE WEIGHTS OF model TO checkpoint[“trained_weights”]
CHANGE THE MODE OF model TO EVALUATION
TURN image TO RGB
RESIZE HEIGHT OF image TO 224
RESIZE WIDTH OF image TO 224
NORMALIZE FIRST CHANNEL OF image WITH A MEAN OF 0.485 AND A STANDARD DEVIATION OF 0.229
NORMALIZE SECOND CHANNEL OF image WITH A MEAN OF 0.456 AND A STANDARD DEVIATION OF 0.224
NORMALIZE THIRD CHANNEL OF image WITH A MEAN OF 0.406 AND A STANDARD DEVIATION OF 0.225
SET prediction TO model(image)
RETURN prediction

**Table 4:** Evaluation metrics for each fold. The last row demonstrates the mean and standard deviation of the final epochs of all folds.

Fold	Total Loss (Cost)	Accuracy	Precision	Recall
1	0.354	0.8906	0.8286	0.9667
2	0.510	0.7812	0.7436	0.8788
3	0.537	0.7656	0.7241	0.7500
4	0.429	0.8438	0.8286	0.8788
5	0.408	0.8254	0.8333	0.8571
Mean ± Standard Deviation	0.447 ± 0.067	0.8213 ± 0.0447	0.7916 ± 0.0476	0.8662 ± 0.0692

## Data Availability

The data presented in this study are available on request from the corresponding author. The data are not publicly available due to privacy restrictions.
